# Supplementary material for: The host RNA polymerase II C-terminal domain is the anchor for replication of the influenza virus genome
Source: Nat Commun. 2024 Feb 5;15:1064. doi: 10.1038/s41467-024-45205-2 (PMC10844641; doi:10.1038/s41467-024-45205-2)
Supplement: Supplementary file 1 — Supplementary Information [file 41467_2024_45205_MOESM1_ESM.pdf]

The host RNA polymerase II C-terminal domain is the anchor for replication of the influenza virus genome

Tim Krischuns[1#*], Benoît Arragain B[#2], Catherine Isel[1], Sylvain Paisant[1], Matthias Budt[3], Thorsten Wolff[3], Stephen Cusack[2*], Nadia Naffakh N[1*]

[1] RNA Biology of Influenza Virus, Institut Pasteur, Université Paris Cité, CNRS UMR 3569, Paris, France

[2] European Molecular Biology Laboratory, Grenoble, France

[3] Unit 17 "Influenza and other Respiratory Viruses", Robert Koch Institut, Berlin, Germany

SUPPLEMENTARY FIGURES S1-S12

FIGURE S1

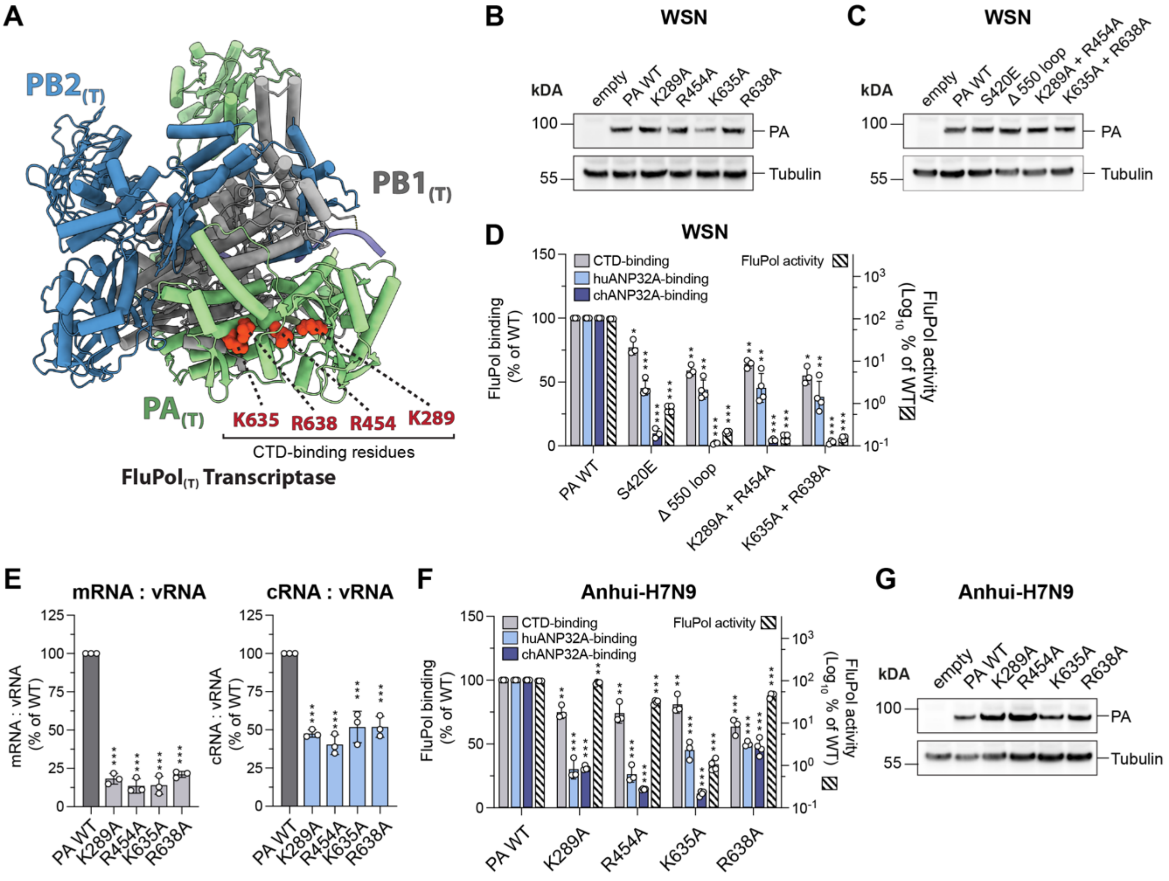

Figure S1: FluPol CTD-binding interface is essential for replication of the viral genome

(A) Ribbon diagram representation of Flu$_A$Pol heterotrimer in the FluPol$_{(T)}$ conformation (A/NT/60/1968, PDB: 6RR7, [https://www.rcsb.org/structure/6rr7] [1]), with PA (green), PB1 (grey), and PB2 (blue). Key FluPol CTD-binding residues are highlighted in red.

(B-C) Lysates of HEK-293T cells transiently expressing for WSN PB1, PB2 and PA with the indicated mutation were analysed by western blot using the indicated antibodies (n=1).

(D) Cell-based WSN FluPol binding and activity assays as in Fig. 1B. Left Y-axis (linear scale): FluPol binding to the CTD, huANP32A and chANP32A. Right Y-axis (logarithmic scale): FluPol activity (hatched bars). Luminescence signals are represented as a percentage of PA WT (mean ± SD, n=3, 4, 3, 4, *p < 0.033, **p < 0.002, ***p < 0.001, one-way ANOVA; Dunnett's multiple comparisons test).

(E) WSN vRNPs with the indicated PA mutations were reconstituted in HEK-293T cells using the NA vRNA segment. Steady-state levels of NA mRNA, cRNA and vRNA were quantified by strand-specific RT-qPCR [2] and are presented as ratios of mRNA to vRNA (grey bars) or cRNA to vRNA (blue bars) levels relative to PA WT (mean ± SD, n=3, ***p < 0.001, one-way ANOVA; Dunnett's multiple comparisons test). RNA levels are presented in Fig. 1C.

(F) Cell-based Anhui-H7N9 FluPol binding and activity assays using a plasmid encoding PA in which the PA-X (PA-ΔX) ORF frame was deleted as described previously [3], as in Fig. 1B. Left Y-axis (linear scale): FluPol binding to the CTD, huANP32A, and chANP32A. Right Y-axis (logarithmic scale): FluPol activity (hatched bars). Luminescence signals are represented as a percentage of PA WT. (mean ± SD, n=3, 3, 3 and 4, **p < 0.002, ***p < 0.001, one-way ANOVA; Dunnett's multiple comparisons test).

(G) Lysates of HEK-293T cells transiently expressing Anhui-H7N9 PB1, PB2 and PA-ΔX with the indicated mutation were analysed by western blot using the indicated antibodies (n=1).

Source data are provided as a Source data file.

FIGURE S2 (1/2)

PB2 Alignment

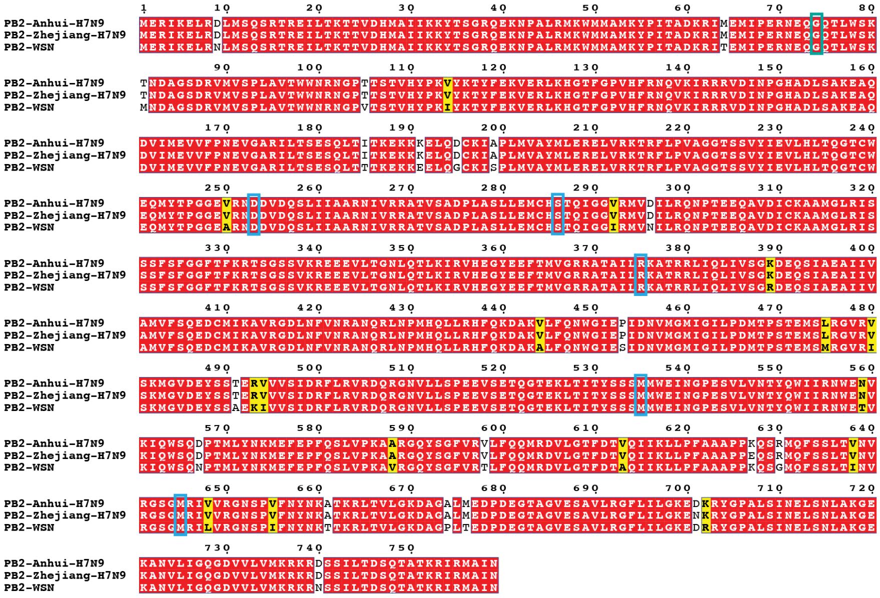

PB1 Alignment

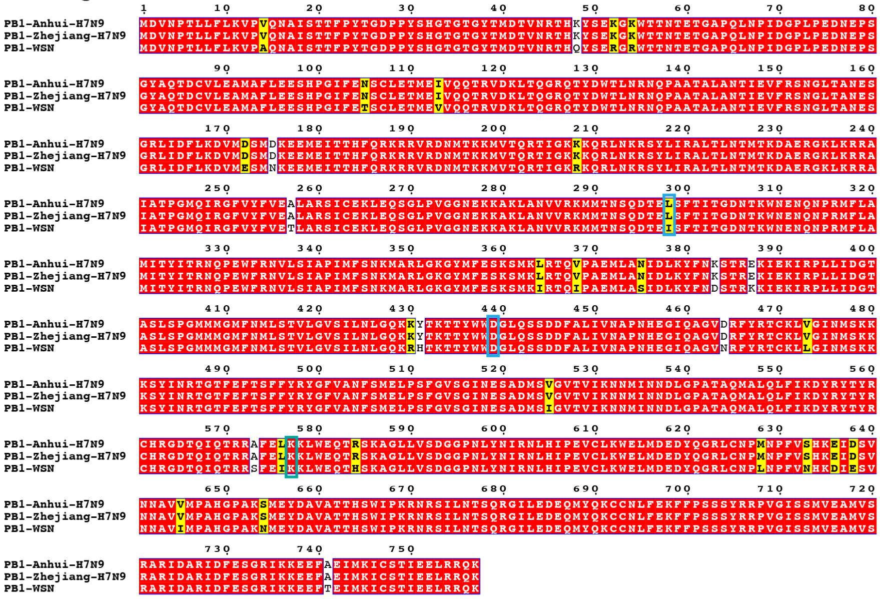

FIGURE S2 (2/2)

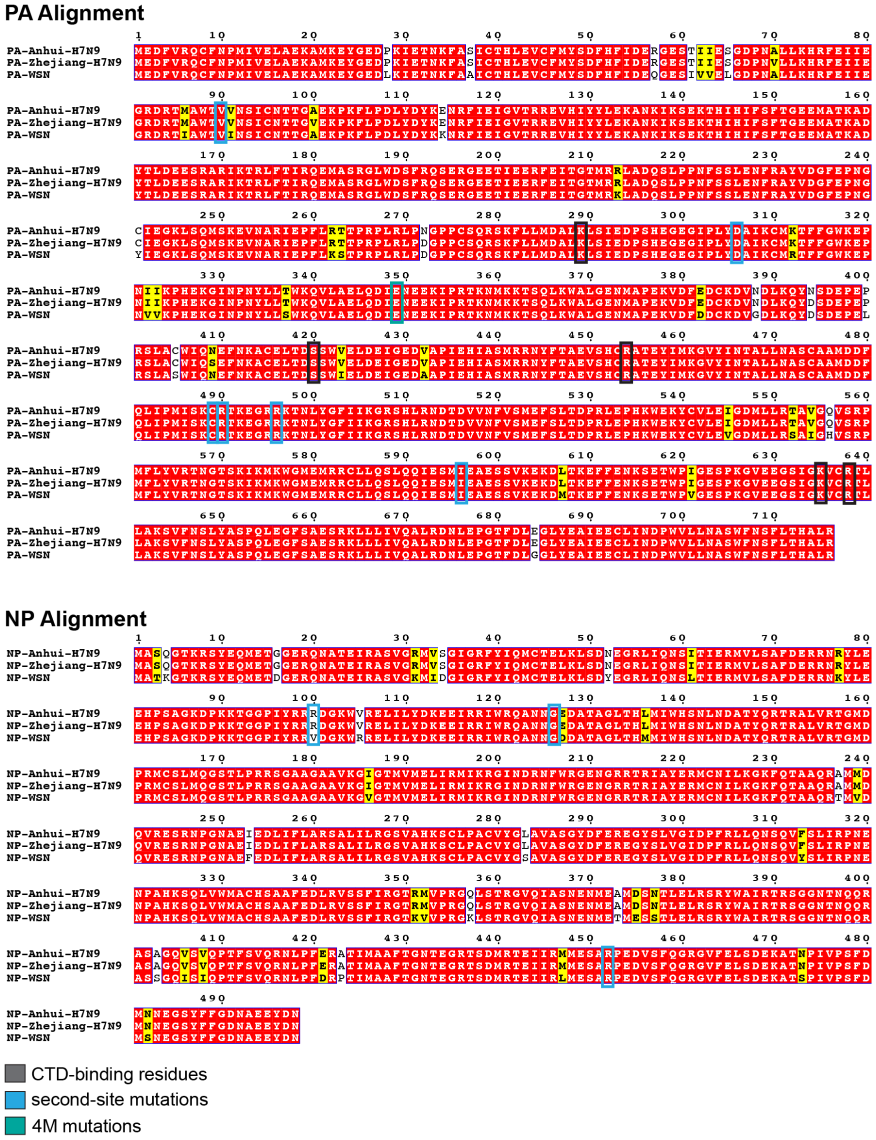

Figure S2: PB2, PB1, PA and NP protein sequence alignments of the influenza A virus strains used in this study

Protein sequences from A/WSN1933(H1N1), A/Anhui/01/2013 (H7N9) and A/Zhejiang/DTID-ZJU01/2013(H7N9) were obtained from UniProt (https://www.uniprot.org/), aligned with CLC Main Workbench 21 and visualised by Espript 3.0 [4]. Identical and similar residues are indicated in red or yellow, respectively. FluPol CTD binding residues subjected to mutagenesis are indicated by grey boxes, FluPol second-site mutations which appeared upon passaging of FluPol CTD-binding mutant viruses by blue boxes and 4M FluPol dimer interrupting residues (PB2 G74, PA E349, PB1 K577) by green boxes.

FIGURE S3

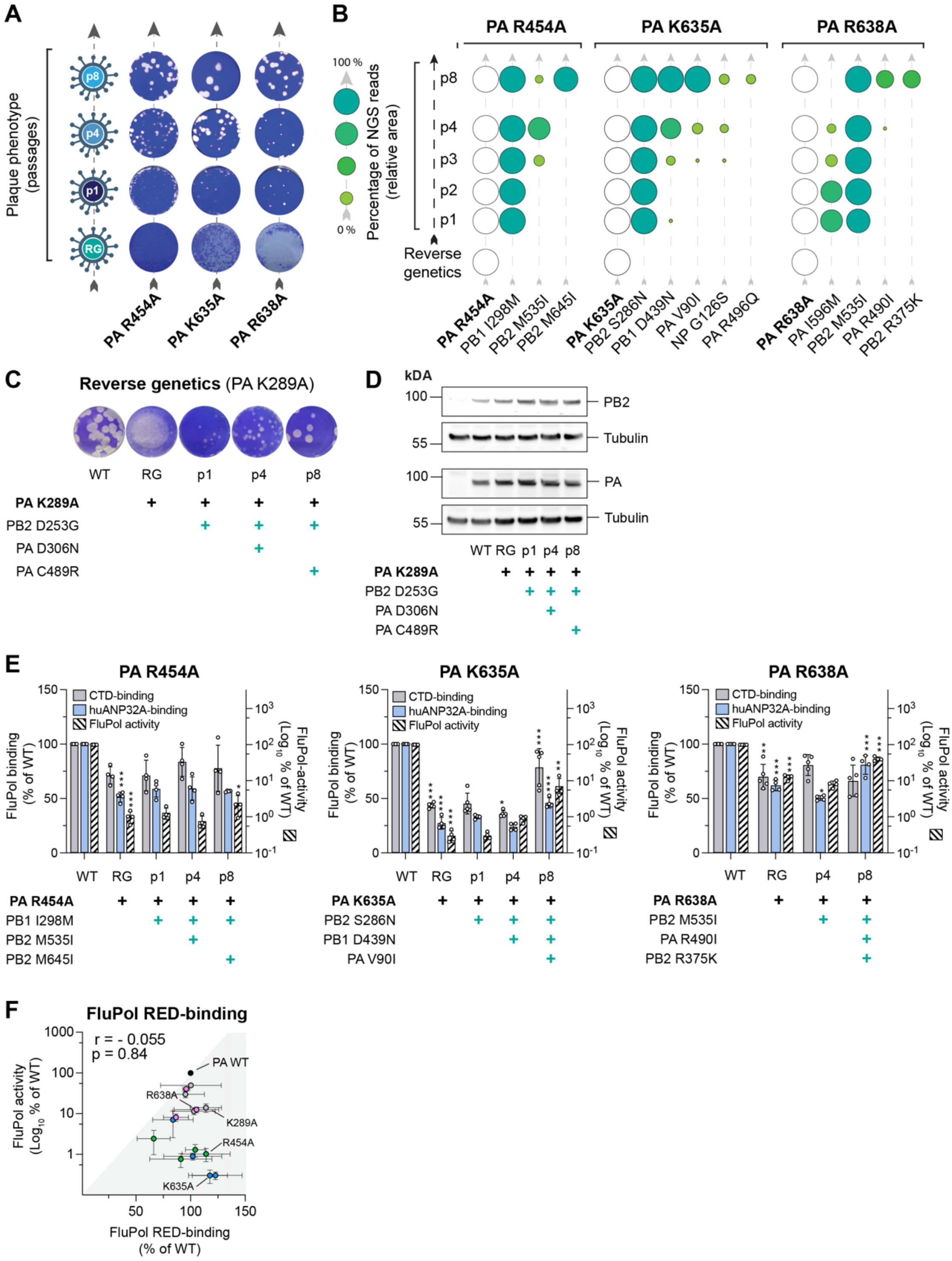

Figure S3: Serial passaging of mutant viruses with mutations at the FluPol-CTD interface selects for adaptive mutations which restore FluPol binding to the CTD and huANP32A

(A) Plaque phenotypes of passaged WSN mutant viruses. Representative images of crystal violet-stained plaque assays on reverse genetics (RG), p1, p4 and p8 supernatants are shown.

(B) Short-read next generation sequencing of the viral genome. Second site mutations found in ≥ 10% of reads in at least one passage are shown (green circles). The fraction of reads showing a given mutation is indicated by

circle area and shades of green (schematic on the left: <25, 25-50, 50-75 and >75 % of reads).
(C) Recombinant viruses with PA K289A and the indicated combinations of second-site mutations were produced by RG and subjected to plaque assay. Representative images of crystal violet-stained plaque assays are shown (n=2).
(D) Lysates of HEK-293T cells transiently expressing WSN PB1, PB2 and PA with the indicated mutation were analysed by western blot using the indicated antibodies (n=1).
(E) Characterisation of combinations of FluPol primary and second-site mutations in cell-based binding and activity assays, as in Fig. 1B. Left Y-axis (linear scale): FluPol binding to the CTD and huANP32A. Right Y-axis (logarithmic scale): FluPol activity (hatched bars). Luminescence signals are represented as a percentage of WT FluPol (mean ± SD, n=5, 4 and 4). Stars indicate statistical significance when passage N is compared to passage N-1, or when RG is compared to WT FluPol (*$p < 0.033$, **$p < 0.002$, ***$p < 0.001$, one-way ANOVA; Tukey's multiple comparisons test).
(F) For each initial FluPol mutant and representative FluPol genotype observed during serial passaging, FluPol binding to RED (mean ± SD, n=4, x-axis) was plotted against FluPol activity (mean ± SD, n=4, y-axis). Combinations of mutations which appeared during passaging of the WSN PA K289A, R454A, K635A and R638A mutant viruses are highlighted in grey, green, blue and pink respectively. r: Pearson correlation coefficient (two-tailed 95% confidence interval).
Source data are provided as a Source data file.

FIGURE S4

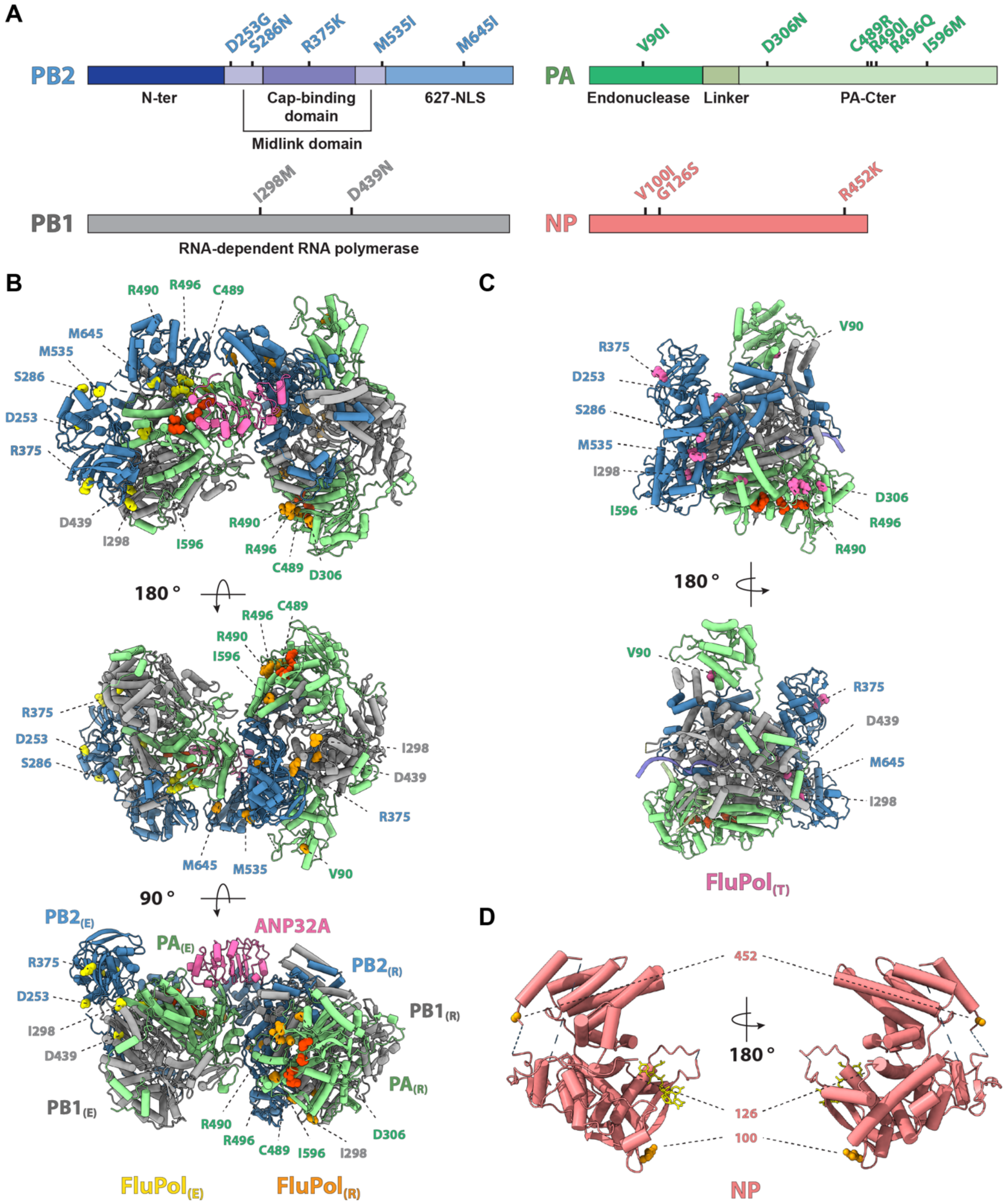

Figure S4: Representation of FluPol and NP second-site mutations which were selected during serial cell culture passaging of PA mutant viruses

(A) Second-site mutations which were selected on FluPol and NP during serial passaging of recombinant plaque-purified PA mutant (K289A, R454A, K635A, R638A) viruses were mapped on a linear domain representation of PB2, PB1, PA and NP. Only second-site mutations found in ≥ 10% of reads in at least one passage are shown.

(B) Model of the Flu$_{A}$Pol replication complex (FluPol$_{(R)}$, FluPol$_{(E)}$ and ANP32A) based on a Flu$_{C}$Pol Cryo-EM structure (C/Johannesburg/1/1966 structure, PDB: 6XZR, [https://www.rcsb.org/structure/6XZR] [5]). Ribbon diagram representation with PA in green, PB1 in grey, PB2 in blue and ANP32A in pink. Initially mutated FluPol residues (PA K289A, R454A, K635A and R638A) are displayed as spheres in red. Residues corresponding to

FluPol second-site mutations were displayed as spheres in orange on the $FluPol_{(R)}$ and in yellow on the $FluPol_{(E)}$, respectively, and are labelled in grey (PB1), blue (PB2) or green (PA).
(C) Representation of the Cryo-EM structure of the FluPol heterotrimer in the $FluPol_{(T)}$ conformation, bound to a 3'5' vRNA promoter and a short capped RNA primer (A/NT/60/1968, PDB: 6RR7 [https://www.rcsb.org/structure/6rr7], [1]). Ribbon diagram representation with PA in green, PB1 in grey, and PB2 in blue. Initially mutated FluPol residues (PA K289A, R454A, K635A and R638A) are displayed as spheres in red and residues corresponding to FluPol second-site mutations in pink.
(D) Residues corresponding to NP second-site mutations were mapped on the RNA-bound crystal structure of a monomeric NP mutant (PDB: 7DXP, [https://www.rcsb.org/structure/7DXP] [6]). Ribbon diagram representation with NP in salmon, RNA in yellow and residues corresponding to NP second-site mutations are displayed as spheres in orange.

FIGURE S5

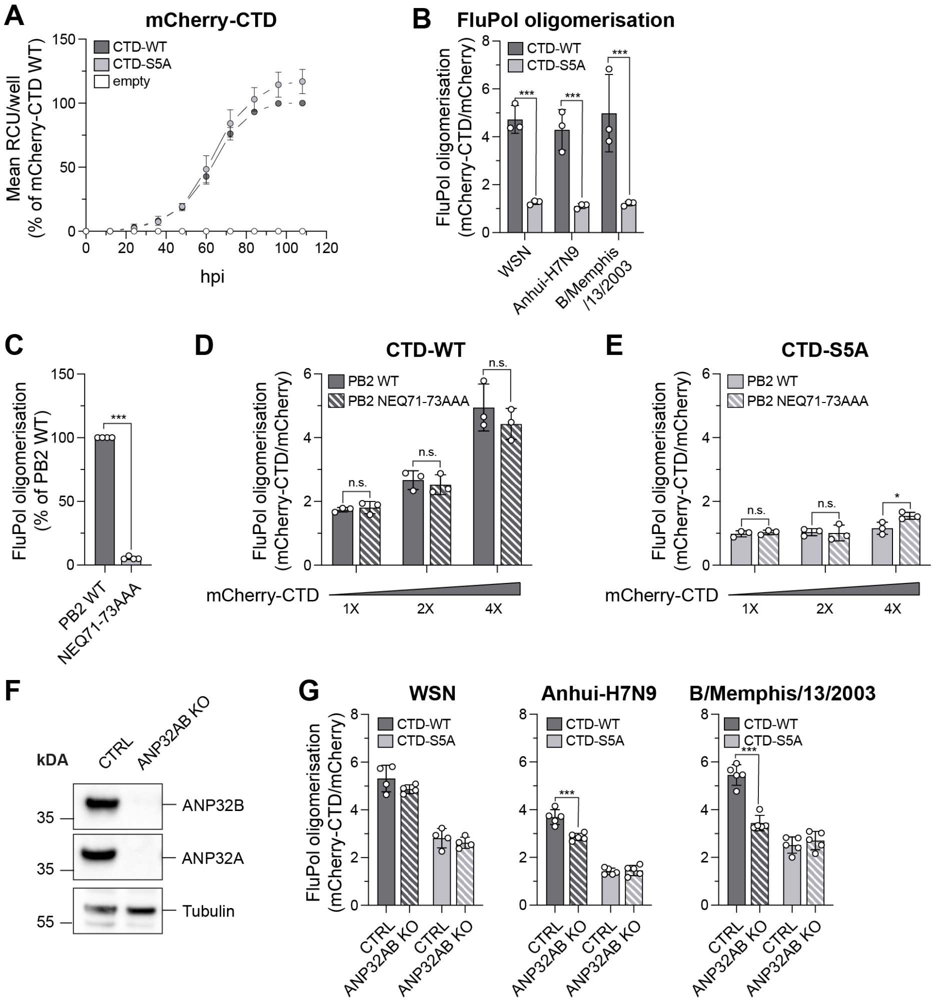

Figure S5: FluPol oligomerisation is enhanced in the presence of CTD and ANP32A

(A) HEK-293T cells were transiently transfected with expression plasmids encoding for mCherry-CTD-WT, mCherry-CTD-S5A or an empty plasmid control. Real-time monitoring of fluorescence was performed by the acquisition of 5 images with a 10X objective per well every 12 h up to 108 hpt. Fluorescence per well (RCU: Red Calibrated Units) is represented as percentages of mCherry-CTD-WT at 108 hpt (mean ± SD, n=6).

(B) WSN, Anhui-H7N9, B/Memphis/13/2003 FluPol oligomerisation was assessed in a split-luciferase-based complementation assay [7]. Untagged mCherry or mCherry tagged to CTD-WT or CTD-S5A (in which serine 5 residues were replaced with alanines) were co-transfected in increasing amounts. Luminescence signals are represented as a percentage of untagged mCherry co-expression. (mean ± SD, n=3, ***p < 0.001, two-way ANOVA; Sidak's multiple comparisons test).

(C) WSN oligomerisation of the FluPol mutant PB2 NEQ71-73AAA was assessed in a split-luciferase-based complementation assay [7]. Luminescence signals are represented as a percentage of PB2 WT. (mean ± SD, n=3, ***p < 0.001, two-tailed 95% confidence interval t-test).

(D-E) WSN FluPol oligomerisation was assessed in a split-luciferase-based complementation assay as in Fig. 3B either with WT FluPol (solid bars) or the PB2 NEQ71-73AAA mutant (hatched bars). Plasmids encoding untagged mCherry or mCherry tagged to (D) CTD-WT or (E) CTD-S5A were co-transfected in increasing amounts. Luminescence signals are represented as fold-changes compared to untagged mCherry co-expression. (mean ± SD, n=3, *p < 0.033, two-way ANOVA; Sidak's multiple comparisons test).

(F) Lysates of HEK-293T control cells (CTRL) and cells in which ANP32A and ANP32B were knocked out by CRISPR-Cas9 (ANP32AB KO) were analysed by western blot using the indicated antibodies (n=1).

(G) FluPol oligomerisation levels of WSN, Anhui-H7N9 and B/Memphis/13/2003 were assessed as described in

Fig. 3B either in HEK-293T CTRL cells (solid bars) or in ANP32AB KO cells (hatched bars). (mean ± SD, n=4, 5, 5, ***p < 0.001 (two-way ANOVA; Sidak's multiple comparisons test).
Source data are provided as a Source data file.

FIGURE S6

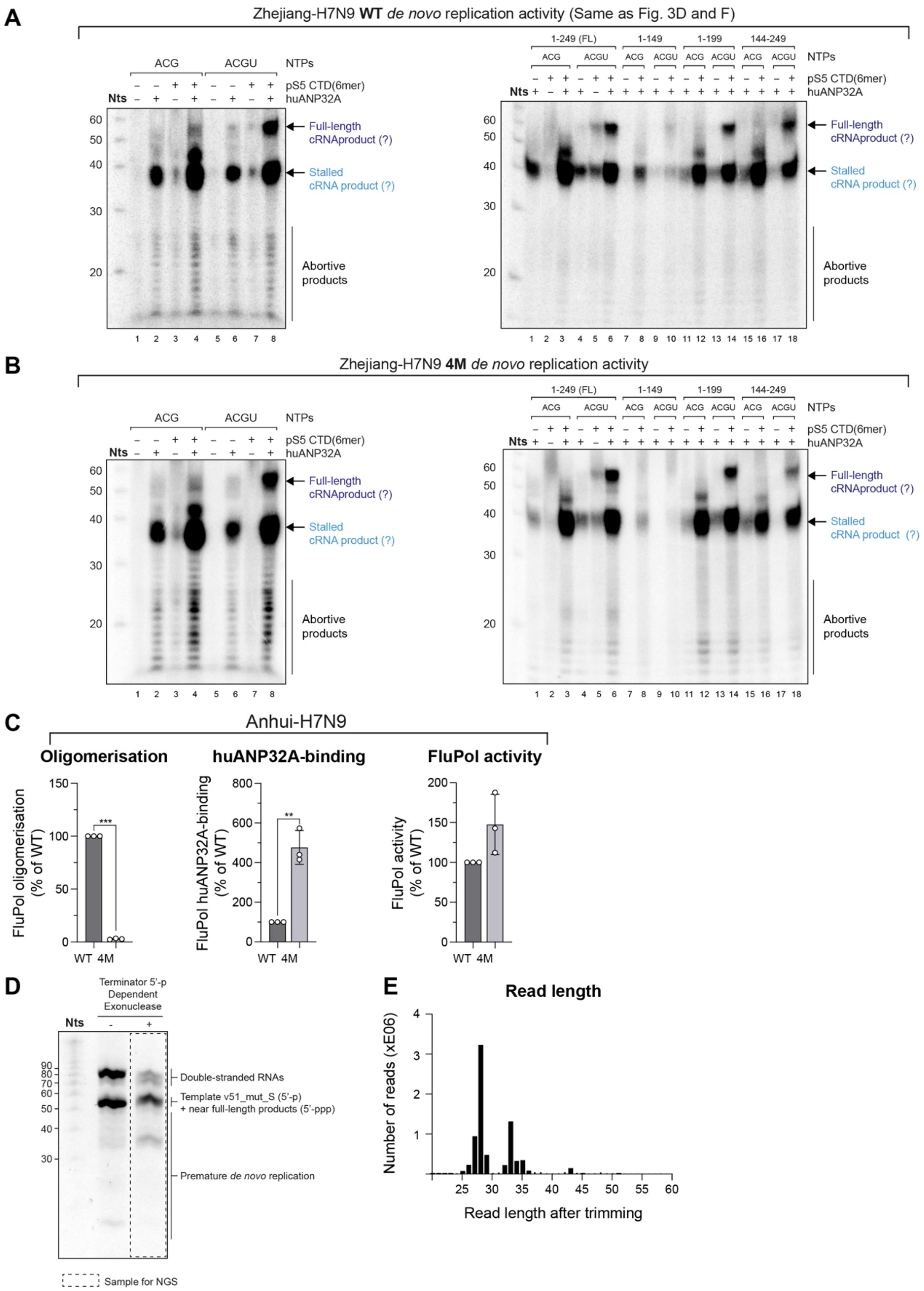

Figure S6: FluPol replication activity is enhanced in the presence of CTD and huANP32A

(A-B) *De novo* replication activity of (A) Zhejiang-H7N9 WT and (B) Zhejiang-H7N9 4M (PA E349K, PA R490I, PB1 K577G, and PB2 G74R) using the v51_mut_S template in the presence of either 3 NTPs (AUG) or 4 NTPs (AUGC), with or without pS5 CTD(6mer) and huANP32A FL (left) or different huANP32A truncation mutants (right) as defined in Fig. 3E. Tentative full-length and stalled replication products are indicated by an

arrow. Nts: molecular weight marker.

(C) Cell-based Anhui-H7N9 WT or 4M FluPol binding and activity assays. FluPol oligomerisation (left) and FluPol binding to huANP32A (middle) was assessed using split-luciferase-based complementation assays. FluPol activity (right) was measured by vRNP reconstitution using a model Fluc-vRNA. Luminescence signals are represented as a percentage of PA WT. (mean ± SD, n=3, $^{**}p < 0.002$, $^{***}p < 0.001$, one-way ANOVA; Dunnett's multiple comparisons test).

(D) NGS sample preparation of FluPol Zheijiang-H7N9 4M *de novo* replication products. Urea-PAGE gel stained with SYBR-Gold of the equivalent *de novo* reaction (Fig. S6B lane 8) before (-) and after (+) 5'-monophosphate (5'-p) RNA digestion using a terminator 5'-p-dependent exonuclease. The dotted rectangle corresponds to the sample sent for NGS. The decade molecular weight marker (Nts) is shown on the left side of the gel.

(E) RNA-sequencing analysis of FluPol Zheijiang-H7N9 4M *de novo* replication products in the presence of pS5 CTD(6mer) and huANP32A. After trimming, the number of reads is plotted according to their lengths.

Source data are provided as a Source data file.

FIGURE S7 (1/2)

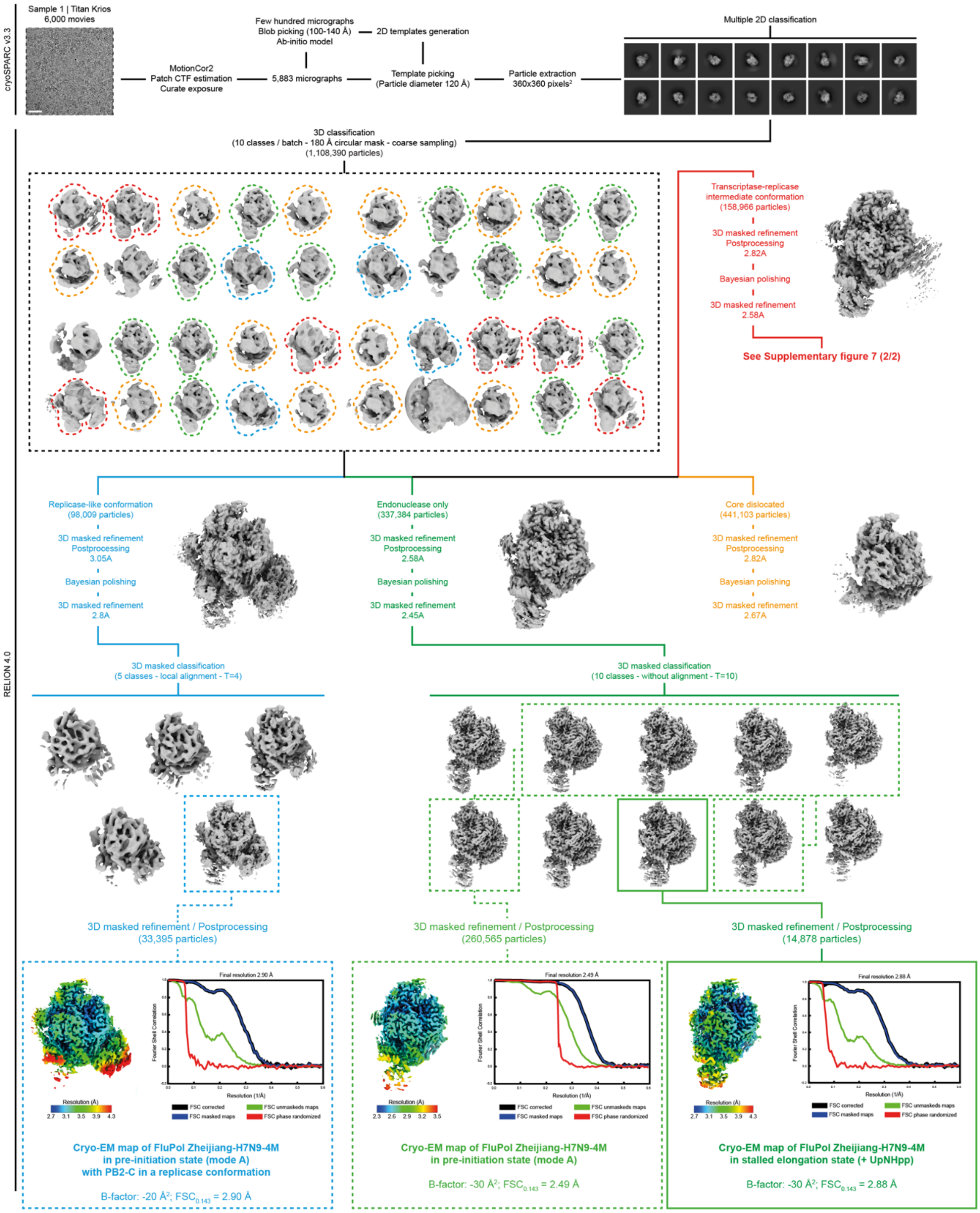
cryoSPARC v3.3
Sample 1 | Titan Krios
6,000 movies
MotionCor2
Patch CTF estimation
Curate exposure
Few hundred micrographs
Blob picking (100-140 Å)
Ab-initio model
2D templates generation
5,883 micrographs
Template picking
(Particle diameter 120 Å)
Particle extraction
360x360 pixels²
Multiple 2D classification
RELION 4.0
3D classification
(10 classes / batch - 180 Å circular mask - coarse sampling)
(1,108,390 particles)
Transcriptase-replicase
intermediate conformation
(158,966 particles)
3D masked refinement
Postprocessing
2.82A
Bayesian polishing
3D masked refinement
2.58A
See Supplementary figure 7 (2/2)
Replicase-like conformation
(98,009 particles)
3D masked refinement
Postprocessing
3.05A
Bayesian polishing
3D masked refinement
2.8A
Endonuclease only
(337,384 particles)
3D masked refinement
Postprocessing
2.58A
Bayesian polishing
3D masked refinement
2.45A
Core dislocated
(441,103 particles)
3D masked refinement
Postprocessing
2.82A
Bayesian polishing
3D masked refinement
2.67A
3D masked classification
(5 classes - local alignment - T=4)
3D masked classification
(10 classes - without alignment - T=10)
3D masked refinement / Postprocessing
(33,395 particles)
3D masked refinement / Postprocessing
(260,565 particles)
3D masked refinement / Postprocessing
(14,878 particles)
Final resolution 2.90 Å
Final resolution 2.49 Å
Final resolution 2.88 Å
Fourier Shell Correlation
Resolution (1/Å)
Resolution (Å)
FSC corrected
FSC masked maps
FSC unmaskeds maps
FSC phase randomized
Cryo-EM map of FluPol Zheijiang-H7N9-4M
in pre-initiation state (mode A)
with PB2-C in a replicase conformation
B-factor: -20 Å²; FSC0.143 = 2.90 Å
Cryo-EM map of FluPol Zheijiang-H7N9-4M
in pre-initiation state (mode A)
B-factor: -30 Å²; FSC0.143 = 2.49 Å
Cryo-EM map of FluPol Zheijiang-H7N9-4M
in stalled elongation state (+ UpNHpp)
B-factor: -30 Å²; FSC0.143 = 2.88 Å

FIGURE S7 (2/2)

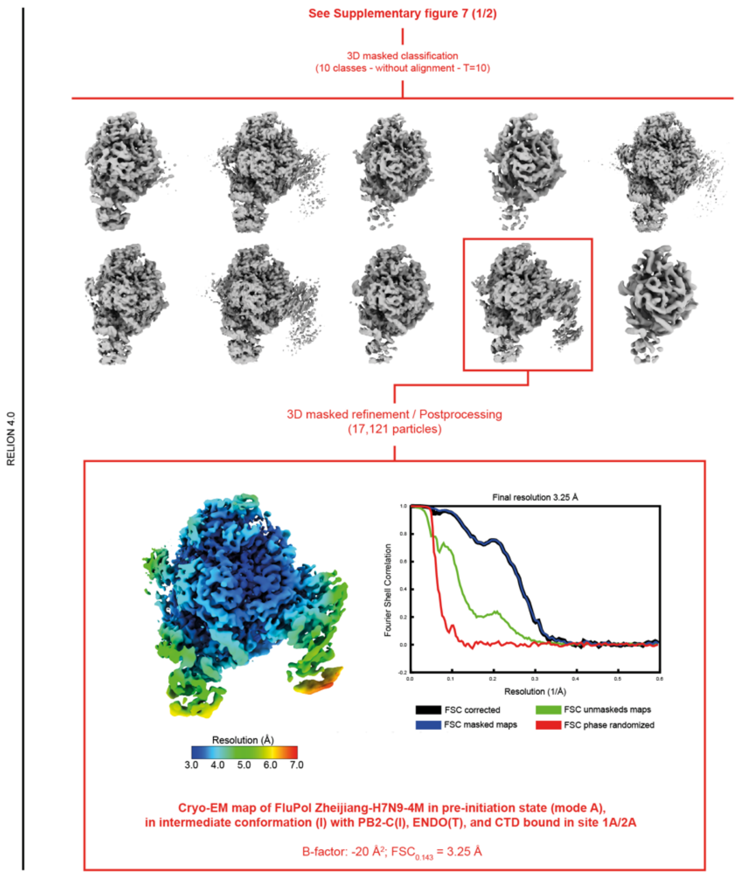

Figure S7: Cryo-EM image processing flowchart to obtain FluPol Zhejiang-H7N9-4M *de novo* replication structures (sample 1| Krios dataset)

Schematics of the image processing strategy used to obtain (i) the Cryo-EM map of FluPol Zhejiang-H7N9-4M in a pre-initiation state (mode A), with PB2-C in a replicase conformation, (ii) the cryo-EM map of FluPol Zhejiang-H7N9-4M in pre-initiation state (mode A) without PB2-C, and (iii) the cryo-EM map of FluPol Zhejiang-H7N9-4M stalled in an elongation state using UpNHpp. Further processing steps for the dislocated FluPols are not shown. Representative cropped micrograph from the TEM Titan Krios dataset, 2D class averages, 3D class averages and intermediate structures are displayed. CTF stands for “Contrast Transfer Function”. The final number of particles, filtered EM maps according to the local resolution and corresponding Fourier Shell Correlation curves (FSC) are shown. Scale bar = 200 Å.

FIGURE S8

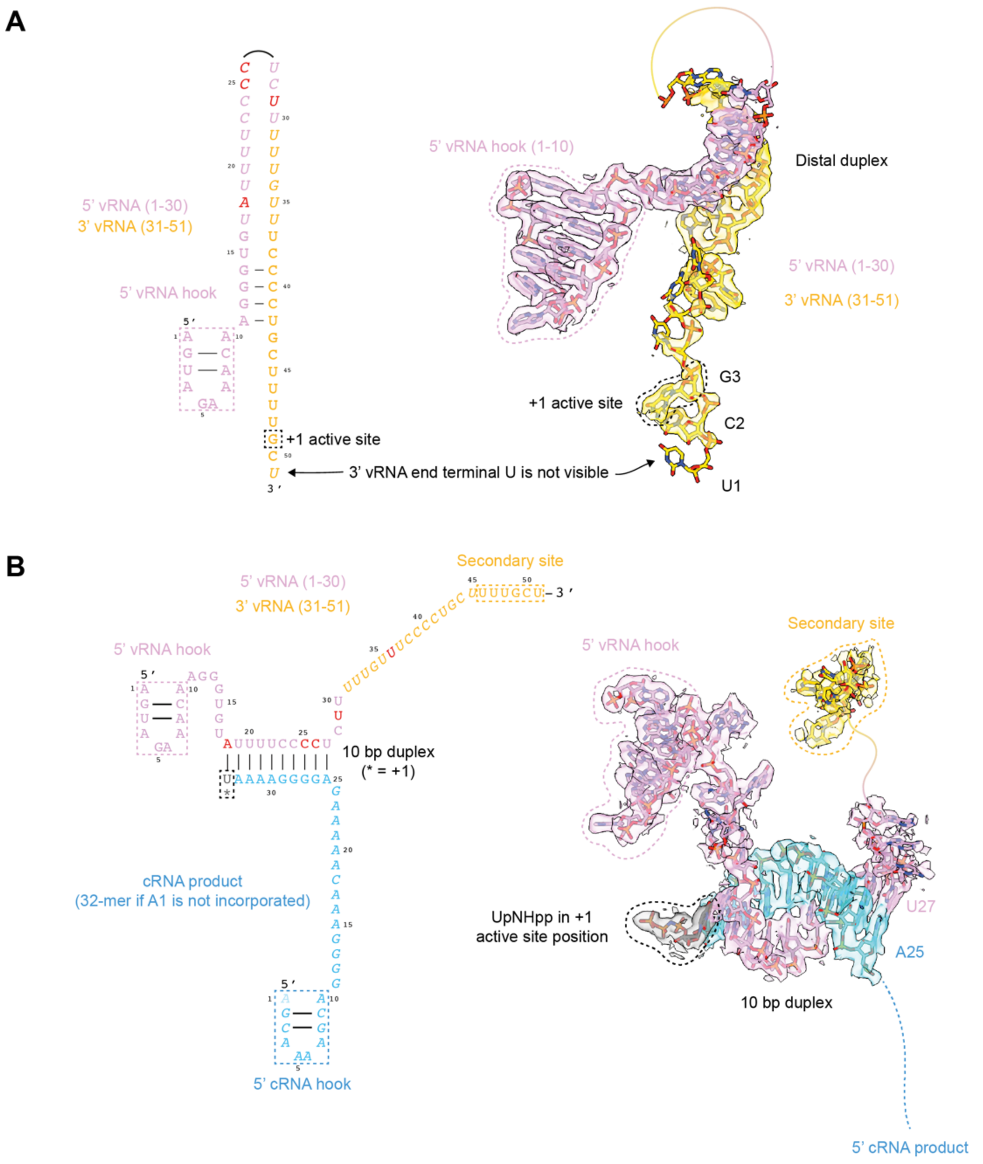

Figure S8: RNA conformation schematics, densities and models of the different states obtained
(A) (Left) Schematics of the v51_mut_S template conformation present in both (i) the cryo-EM map of FluPol Zhejiang-H7N9-4M in pre-initiation state (mode A) without PB2-C or (ii) with PB2-C in replicase conformation. The 5' vRNA end (1-30) is coloured in pink. The 3' vRNA end (31-51) is coloured in gold. Introduced mutations are coloured in red. Unseen nucleotides are in italics. The +1 active site position is indicated by a dotted rectangle. (Right) Coulomb potential of the v51_mut_S isolated from the cryo-EM map of FluPol Zhejiang-H7N9 4M in pre-initiation state (mode A) without PB2-C, at an overall resolution of 2.5 Å. 3'-U1 is displayed but not visible in the cryo-EM map. 3'-G3 is in the +1 active site position.
(B) (Left) Schematics of the v51_mut_S template and the *de novo* cRNA replication product (33-mer) conformations present in the cryo-EM map of FluPol Zhejiang-H7N9-4M stalled in an elongation state using UpNHpp. The template is coloured as in A. The *de novo* cRNA product is coloured in blue. Unseen nucleotides are in italics. The +1 active site position is indicated. (Right) Coulomb potential of the v51_mut_S template and the *de novo* cRNA replication product (33-mer, 32-mer if A1 is not incorporated) isolated from the cryo-EM map of FluPol Zhejiang-H7N9-4M stalled in an elongation state using UpNHpp, at an overall resolution of 2.9 Å. The non-hydrolysable UpNHpp is in the +1 active site position, coloured in dark grey and circled by a dotted line. Flexible nucleotides are shown as dotted lines.

FIGURE S9

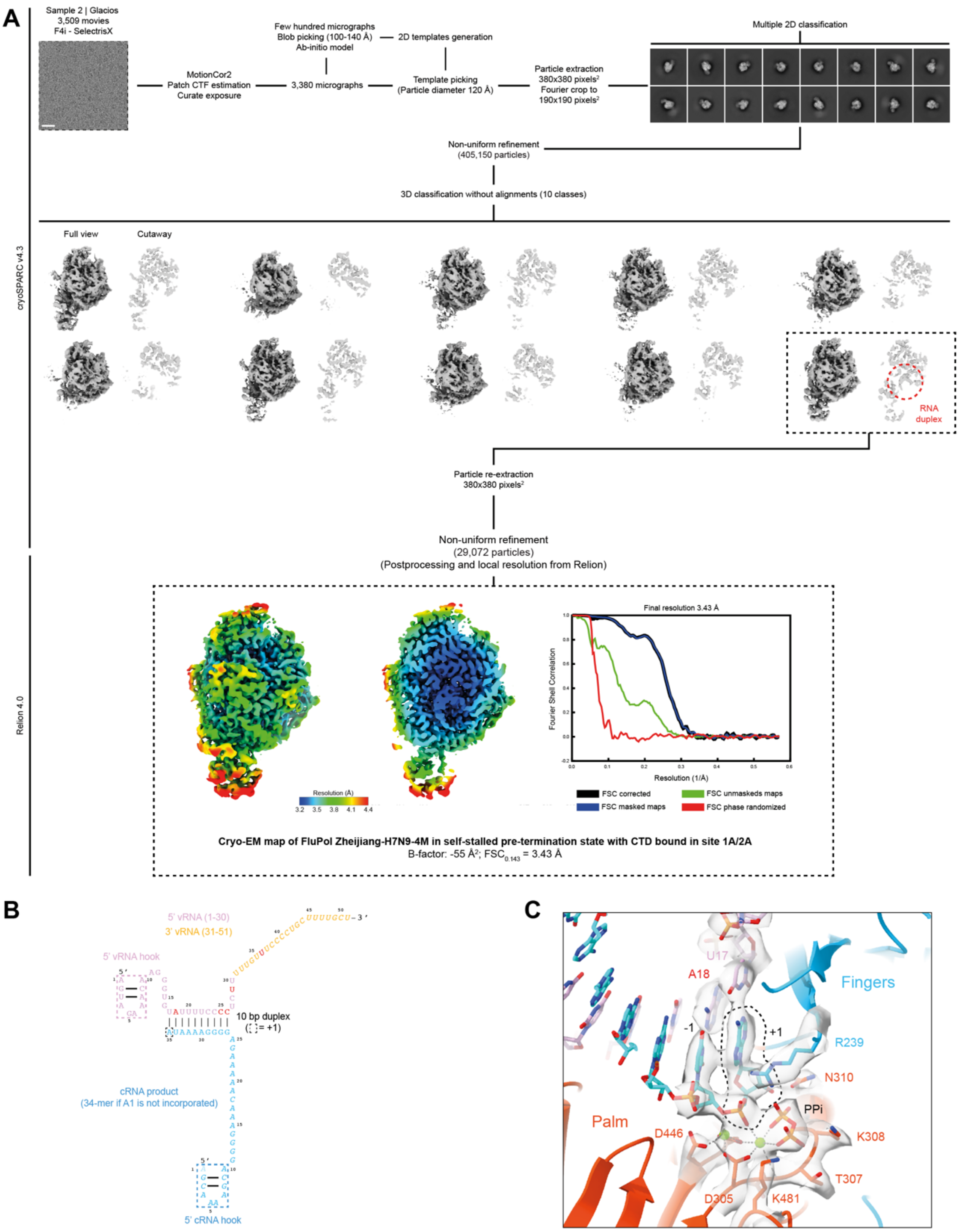

Figure S9: Cryo-EM image processing flowchart to obtain FluPol Zhejiang-H7N9-4M self-stalled elongation structure (sample 2 | Glacios)

(A) Schematics of the image processing strategy used to obtain the cryo-EM map of FluPol Zhejiang-H7N9 in self-stalled elongation state. Representative cropped micrograph from the TEM Glacios dataset, 2D class averages, 3D class averages are displayed. CTF stands for “Contrast Transfer Function”. The final number of particles, filtered EM maps according to the local resolution and corresponding Fourier Shell Correlation curves (FSC) are shown. Scale bar = 200 Å.

(B) Schematics of the v51_mut_S template and the *de novo* cRNA replication product (35-mer or 34-mer if A1 is not incorporated) conformations present in the cryo-EM map of FluPol Zhejiang-H7N9-4M self-stalled in

elongation state. The 5’ vRNA end (1-30) is coloured in pink. The 3’ vRNA end (31-51) is coloured in gold. Introduced mutations are coloured in red. Unseen nucleotides are in italics. The +1 active site position is indicated by a dotted rectangle. The *de novo* cRNA product is coloured in blue. Unseen nucleotides are in italics.

(C) Close-up view on the template-product RNA duplex in FluPol Zhejiang-H7N9-4M self-stalled pre-termination state structure. The Coulomb potential map of the template (up to A18), the *de novo* cRNA product (+1/-1 active site positions only), Mg^{2+} ions, pyrophosphate (PPi), and key active site residues is shown. The RNA is coloured as in B. The +1 active site position is highlighted by a dotted line. The palm domain is coloured in orange. Key residues of the active site are displayed. Mg^{2+} ions are coloured in green. The finger domain is coloured in blue.

FIGURE S10

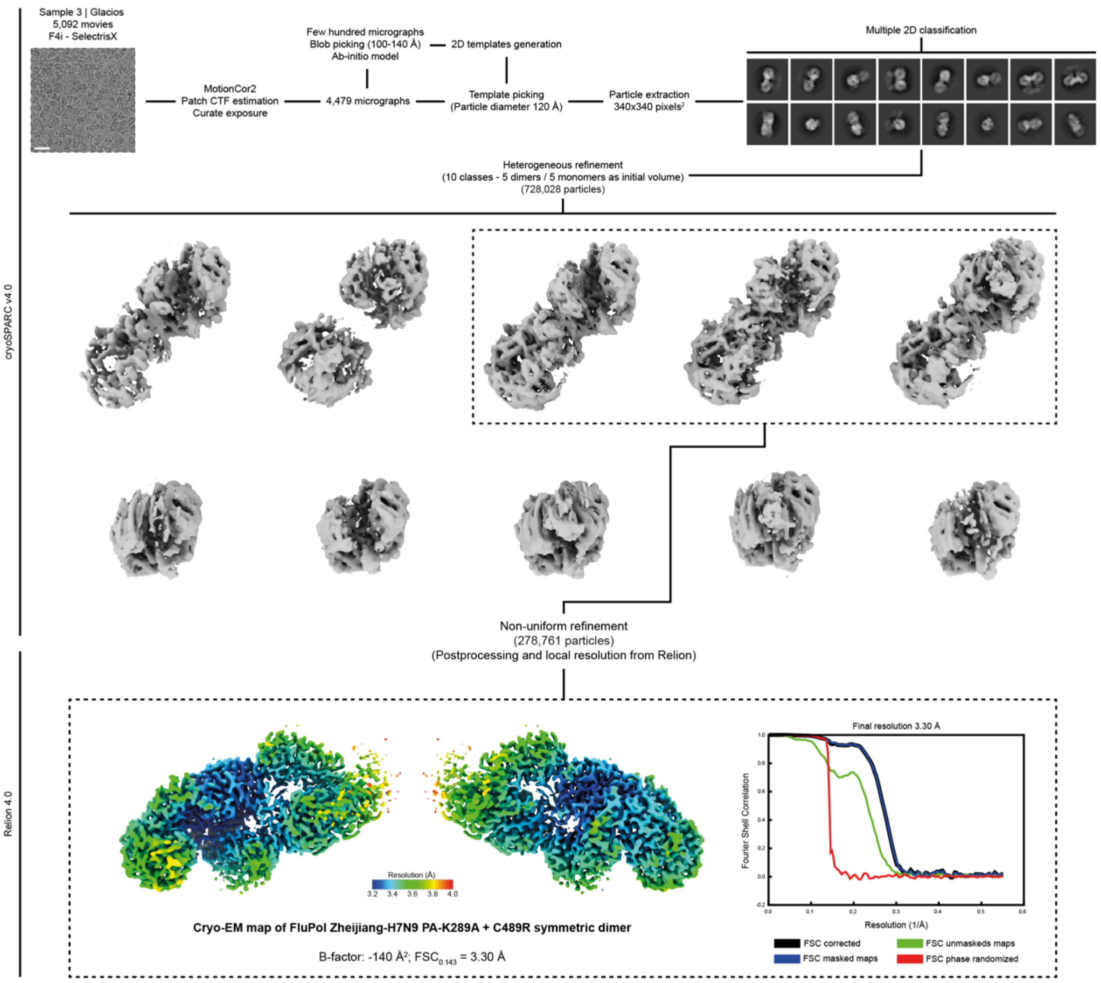

Figure S10: Cryo-EM image processing flowchart to obtain FluPol Zhejiang-H7N9 PA K289A+C489R structure (sample 3 | Glacios)

Schematics of the image processing strategy used to obtain the cryo-EM map of FluPol Zhejiang-H7N9 PA K289A+C489R. Representative cropped micrograph from the TEM Glacios dataset, 2D class averages, 3D class averages and intermediate structures are displayed. CTF stands for “Contrast Transfer Function”. The final number of particles, filtered EM maps according to the local resolution and corresponding Fourier Shell Correlation curves (FSC) are shown. Scale bar = 200 Å.

FIGURE S11

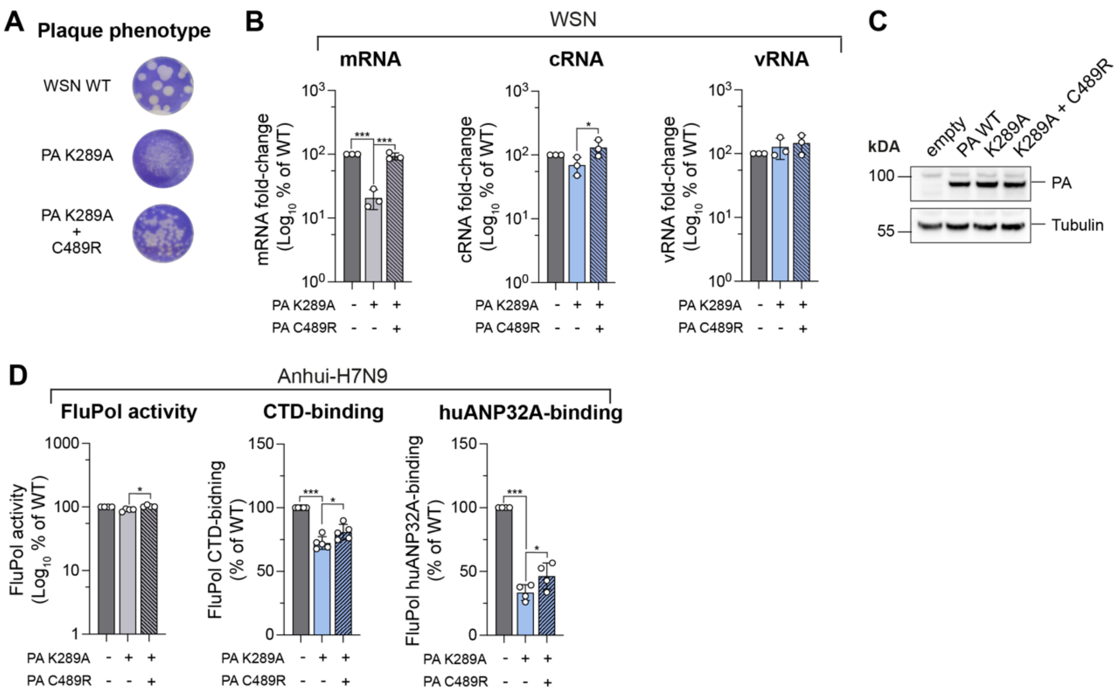

Figure S11: Restoration of CTD-binding Site 2A rescues FluPol replication activity and enhances FluPol binding to huANP32A

(A) Recombinant WSN mutant viruses were produced by reverse genetics. Representative plaque assays as quantified in Fig. 5D of mutant viruses harbouring PA K289A and the second-site mutation PA C489R are shown. The supernatants of RG experiments (n=2) were titrated on MDCK cells and stained by crystal violet.
(B) WSN vRNPs with the PA K289A mutation or the double mutation PA K289A+C489R were reconstituted in HEK-293T cells by transient transfection using a plasmid encoding the NA vRNA segment. Steady-state levels of NA mRNA, cRNA and vRNA were quantified by strand-specific RT-qPCR [2] normalised to GAPDH by the $2^{-\Delta\Delta CT}$ method [8] and are represented as a percentage of PA WT. (mean ± SD, n=3, *p < 0.033, **p < 0.002, ***p < 0.001, one-way ANOVA; Dunnett's multiple comparisons test).
(C) HEK-293T cells were co-transfected with expression plasmids for WSN PB1, PB2 and PA with the indicated mutation. Cell lysates were analysed by western blot using antibodies specific for PA and tubulin (n=1).
(D) Anhui-H7N9 FluPol with the PA K289A mutation and the second-site mutation PA C489R were characterised. (left): Anhui-H7N9 FluPol activity was measured by vRNP reconstitution (PB2, PB1, PA-ΔX, NP) in HEK-293T cells, using a model vRNA encoding the Firefly luciferase. Luminescence was measured and normalised to a transfection control. The data are represented as a percentage of PA WT. (middle): Anhui-H7N9 FluPol binding to the CTD was assessed using a split-luciferase-based complementation assay. HEK-293T cells were co-transfected with expression plasmids for the CTD tagged with one fragment of the *G. princeps* luciferase and FluPol tagged with the other fragment. Luminescence due to luciferase reconstitution was measured and the data are represented as a percentage of PA WT. (right): huANP32A was tagged with one fragment of the *G. princeps* luciferase and binding to Anhui-H7N9 FluPol was determined as for FluPol binding to the CTD. (mean ± SD, n=4, 5, 4, *p < 0.033, ***p < 0.001, one-way ANOVA; Dunnett's multiple comparisons test).
Source data are provided as a Source data file.

FIGURE S12

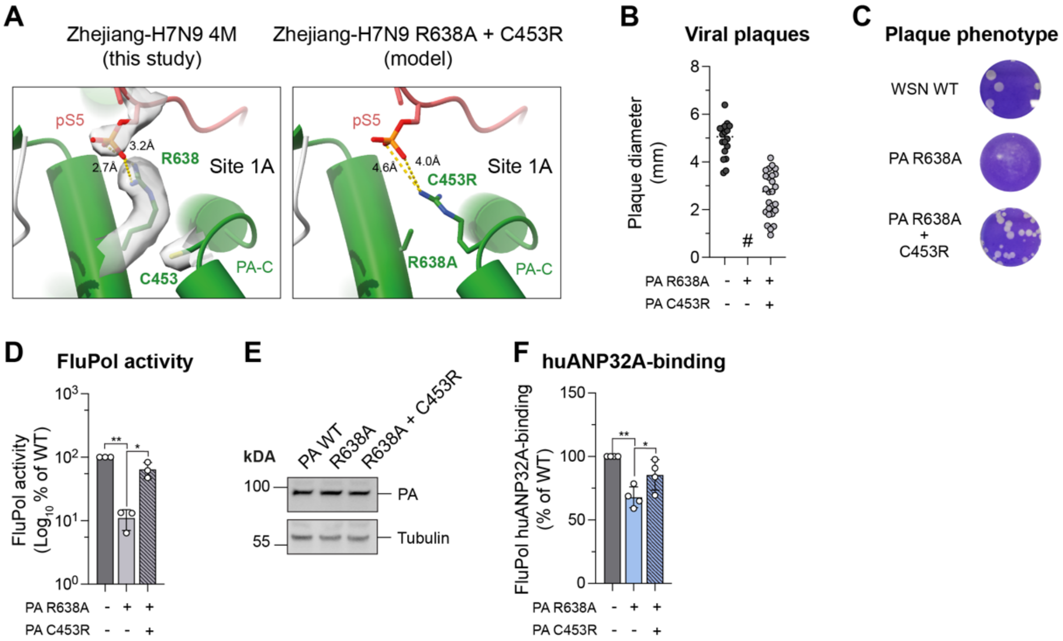

Figure S12: Restoration of CTD-binding Site 1 rescues FluPol replication activity and enhances FluPol binding to huANP32A

(A) (left) Cartoon representation of the pS5 CTD bound to FluPol Zhejiang-H7N9 4M (replicase-like conformation) in CTD-binding site 1A. Green: PA subunit; Red: pS5 CTD; yellow dashed lines : putative hydrogen bonds between PA R638 and pS5 (corresponding distances are indicated). The Coulomb potential map of PA C453, PA R638, and pS5 is shown. (right) Model derived from FluPol Zhejiang-H7N9 4M structure bearing PA C453R and PA R638A mutations. Green: PA subunit; red: pS5 CTD. PA C453R and PA R638A residues are displayed. Hypothetical distances between PA C453R and pS5 are shown.

(B-F) Phenotypes associated with the WSN FluPol PA R638A mutation and the second-site mutation PA C453R. (B-C) Plaque phenotype of recombinant WSN mutant viruses produced by reverse genetics (n=2). RG supernatants were titrated on MDCK cells and stained by crystal violet. Plaque diameters (mm) were measured and each dot represents one viral plaque. Representative plaque assays are shown in (C) (#) pinhead-sized plaques. (D) WSN FluPol activity was measured by vRNP reconstitution in HEK-293T cells using a model Fluc-vRNA. Luminescence signals are represented as a percentage of PA WT (mean ± SD, n=3). (E) HEK-293T cells were co-transfected with expression plasmids for WSN PB1, PB2 and PA with the indicated mutation. Cell lysates were analysed by western blot using the indicated antibodies (n=1). (F) WSN FluPol binding to the huANP32A was assessed using a split-luciferase-based complementation assay. Luminescence signals are represented as a percentage of PA WT. (mean ± SD, n=4, $*p < 0.033$, $**p < 0.002$, one-way ANOVA; Dunnett's multiple comparisons test).

Source data are provided as a Source data file.

References

1. Fan, H. *et al.* Structures of influenza A virus RNA polymerase offer insight into viral genome replication. *Nature* **573**, 287–290 (2019).
2. Kawakami, E. *et al.* Strand-specific real-time RT-PCR for distinguishing influenza vRNA, cRNA, and mRNA. *J Virol Methods* **173**, 1–6 (2011).
3. Jagger, B. W. *et al.* An overlapping protein-coding region in influenza A virus segment 3 modulates the host response. *Science* **337**, 199–204 (2012).
4. Robert, X. & Gouet, P. Deciphering key features in protein structures with the new ENDscript server. *Nucleic Acids Res* **42**, W320-324 (2014).
5. Carrique, L. *et al.* Host ANP32A mediates the assembly of the influenza virus replicase. *Nature* **587**, 638–643 (2020).
6. Tang, Y.-S., Xu, S., Chen, Y.-W., Wang, J.-H. & Shaw, P.-C. Crystal structures of influenza nucleoprotein complexed with nucleic acid provide insights into the mechanism of RNA interaction. *Nucleic Acids Res* **49**, 4144–4154 (2021).
7. Chen, K.-Y., Santos Afonso, E. D., Enouf, V., Isel, C. & Naffakh, N. Influenza virus polymerase subunits co-evolve to ensure proper levels of dimerization of the heterotrimer. *PLoS Pathog* **15**, e1008034 (2019).
8. Livak, K. J. & Schmittgen, T. D. Analysis of relative gene expression data using real-time quantitative PCR and the 2(-Delta Delta C(T)) Method. *Methods* **25**, 402–408 (2001).
