# Supplementary material for: The host RNA polymerase II C-terminal domain is the anchor for replication of the influenza virus genome
Source: Nat Commun. 2024 Feb 5;15:1064. doi: 10.1038/s41467-024-45205-2 (PMC10844641; doi:10.1038/s41467-024-45205-2)
Supplement: Supplementary file 3 — Reporting Summary [file 41467_2024_45205_MOESM3_ESM.pdf]

nature portfolio

Corresponding author(s): Tim Krischuns
Stephen Cusack
Nadia Naffakh

Last updated by author(s): Nov 23, 2023

Reporting Summary

Nature Portfolio wishes to improve the reproducibility of the work that we publish. This form provides structure for consistency and transparency in reporting. For further information on Nature Portfolio policies, see our Editorial Policies and the Editorial Policy Checklist.

Statistics

For all statistical analyses, confirm that the following items are present in the figure legend, table legend, main text, or Methods section.

n/a	Confirmed	
☐	☒	The exact sample size (*n*) for each experimental group/condition, given as a discrete number and unit of measurement
☐	☒	A statement on whether measurements were taken from distinct samples or whether the same sample was measured repeatedly
☐	☒	The statistical test(s) used AND whether they are one- or two-sided<br>*Only common tests should be described solely by name; describe more complex techniques in the Methods section.*
☒	☐	A description of all covariates tested
☐	☒	A description of any assumptions or corrections, such as tests of normality and adjustment for multiple comparisons
☐	☒	A full description of the statistical parameters including central tendency (e.g. means) or other basic estimates (e.g. regression coefficient) AND variation (e.g. standard deviation) or associated estimates of uncertainty (e.g. confidence intervals)
☐	☒	For null hypothesis testing, the test statistic (e.g. *F*, *t*, *r*) with confidence intervals, effect sizes, degrees of freedom and *P* value noted<br>*Give P values as exact values whenever suitable.*
☒	☐	For Bayesian analysis, information on the choice of priors and Markov chain Monte Carlo settings
☒	☐	For hierarchical and complex designs, identification of the appropriate level for tests and full reporting of outcomes
☐	☒	Estimates of effect sizes (e.g. Cohen's *d*, Pearson's *r*), indicating how they were calculated

Our web collection on statistics for biologists contains articles on many of the points above.

Software and code

Policy information about availability of computer code

Data collection	MikroWin Version 4.41; Light Cycler Software 1.5.0.39; Image Lab Touch Software 2.4.0.03; Amersham Typhoon Scanner 3.0
Data analysis	GraphPad Prism Version 9.5.1; - ImageLab Biorad Version 6.01 build 34; - ImageJ2 Version 2.3.0/1.3q; - Cutadapt v 2.3; - Relion's Motioncor implementation; - Relion v4.0t; - cryoSPARC v 3.3; - COOT version 0.9.8; - Phenix version 1.20.1; - Chimera X version 1.6.1

For manuscripts utilizing custom algorithms or software that are central to the research but not yet described in published literature, software must be made available to editors and reviewers. We strongly encourage code deposition in a community repository (e.g. GitHub). See the Nature Portfolio guidelines for submitting code & software for further information.

Data

Policy information about availability of data

All manuscripts must include a data availability statement. This statement should provide the following information, where applicable:

- Accession codes, unique identifiers, or web links for publicly available datasets
- A description of any restrictions on data availability
- For clinical datasets or third party data, please ensure that the statement adheres to our policy

The coordinates and EM maps generated in this study have been deposited in the Protein Data Bank and the Electron Microscopy Data Bank:
Infuenza A/Zhejiang-H7N9 polymerase (4M mutant) in replicase-like conformation in pre-initiation state with RNAP II pS5 CTD peptide mimic bound in site 1A/2A, PDB 8PM0 [https://www.rcsb.org/structure/8pm0] and EMD-17755;

Influenza A/Zhejiang-H7N9 polymerase (4M mutant) in pre-initiation state with continuous RNAP II pS5 CTD peptide mimic bound in site 1A/2A PDB 8PNP [https://www.rcsb.org/structure/8pnp] and EMD-17782;
Influenza A/Zhejiang-H7N9 polymerase (4M mutant) in elongation state with continuous RNAP II pS5 CTD peptide mimic bound in site 1A/2A PDB 8PNQ [https://www.rcsb.org/structure/8pnq] and EMD-17783;
Influenza A/Zhejiang-H7N9 polymerase (PA K289A+C489R) symmetric dimer bound to the promoter PDB 8POH [https://www.rcsb.org/structure/8poh] and EMD-17792.
Influenza A/H7N9 polymerase in pre-initiation state, intermediate conformation (I) with PB2-C(I), ENDO(T), and Pol II pS5 CTD peptide mimic bound in site 1A/2A PDB 8R3L [https://www.rcsb.org/structure/8r3l] and EMD-18872;
Influenza A/H7N9 polymerase in self-stalled pre-termination state, with Pol II pS5 CTD peptide mimic bound in site 1A/2A PDB 8R3K [https://www.rcsb.org/structure/8r3k] and EMD-18871.
The NGS raw reads generated in this study have been deposited in the European nucleotide archive under accession code ERP149587 [https://www.ebi.ac.uk/ena/browser/view/PRJEB64419].
The sequence files of plasmids used in this study have been deposited in the Zenodo repository [https://doi.org/10.5281/zenodo.10462746].
The raw data generated in this study are provided in the Source Data File.

Research involving human participants, their data, or biological material

Policy information about studies with human participants or human data. See also policy information about sex, gender (identity/presentation), and sexual orientation and race, ethnicity and racism.

Reporting on sex and gender	N/A
Reporting on race, ethnicity, or other socially relevant groupings	N/A
Population characteristics	N/A
Recruitment	N/A
Ethics oversight	N/A

Note that full information on the approval of the study protocol must also be provided in the manuscript.

Field-specific reporting

Please select the one below that is the best fit for your research. If you are not sure, read the appropriate sections before making your selection.

☒ Life sciences ☐ Behavioural & social sciences ☐ Ecological, evolutionary & environmental sciences

For a reference copy of the document with all sections, see nature.com/documents/nr-reporting-summary-flat.pdf

Life sciences study design

All studies must disclose on these points even when the disclosure is negative.

Sample size	Sample sizes were estimated on the basis of previous studies using similar methods and analyses that are widely published, for example see PMID: 31694956, PMID: 36774438, PMID: 31581279, PMID: 32304664.
Data exclusions	No data were excluded from the analysis
Replication	Cell-based experiments using luminescence or RTqPCR assays and de novo replication assays using purified proteins were performed with at least three independent biological replicates, with all attempts at replication being successful.<br>Protein expression analyses by western-blot were performed once.
Randomization	Randomization is not relevant to this study, given that samples were not assigned to any groups.
Blinding	No blinding was performed as the experiments were performed and analyzed by the same person.

Reporting for specific materials, systems and methods

We require information from authors about some types of materials, experimental systems and methods used in many studies. Here, indicate whether each material, system or method listed is relevant to your study. If you are not sure if a list item applies to your research, read the appropriate section before selecting a response.

Materials & experimental systems

n/a	Involved in the study
	☒ Antibodies
	☒ Eukaryotic cell lines
☒	Palaeontology and archaeology
☒	Animals and other organisms
☒	Clinical data
☒	Dual use research of concern
☒	Plants

Methods

n/a	Involved in the study
☒	ChIP-seq
☒	Flow cytometry
☒	MRI-based neuroimaging

Antibodies

Antibodies used

pS5 CTD (clone 3E8, Active Motif, lot number 10618002, 1:1000)
https://www.activemotif.com/catalog/details/61085
Validation data included in the manuscript: upon western blot analysis, lysates from control mock-transfected cells & cells transfected with the S5A-CTD mutant plasmid do not show the expected band migrating at 120 kDa, while it is observed for cells transfected with the pS5 CTD plasmid as previously published, (see PMID: 35605026, Fig. 2F).

mCherry (26765-1-AP, Proteintech, 1:1000)
https://www.ptglab.com/products/mCherry-Antibody-26765-1-AP.htm
Validation data included in the manuscript: upon western blot analysis, lysates from control mock-transfected cells do not show the expected band migrating at 110-120 kDa, while it is observed for cells transfected with the mCherry plasmid.

V5 (SV5-Pk1, Thermo Fisher, lot numbers 1242533, 2534913, 1:5000)
https://www.thermofisher.com/antibody/product/V5-Tag-Antibody-Monoclonal/R960-25
Validation data included in the manuscript: upon western blot analysis, lysates from control mock-transfected cells do not show bands, while it is observed for cells transfected with the ANP32A-V5 plasmid showing the expected band at 40 kDa.

Tubulin (B-5-1-2, Sigma Aldrich, lot number 0000089499, 1:10000)
https://www.sigmaaldrich.com/FR/en/product/sigma/t5168
Validation data shown on the provider's website: upon western blot analysis, lysates from control tubulin-negative control worms do not show the expected band, while it is observed for tubulin-expressing worms (Figure 8E, Solinger et al. PLOS Genetics 2008, DOI: 10.1371/journal.pgen.1000820)

PA (Da Costa et al., 2015 PMID: 25855727, 1:2500)
A rabbit serum directed against the PA domain (residues 197 to 257) was used to reveal PA expression.
Validation data included in the manuscript: upon western blot analysis, lysates from control mock-transfected cells do not show the expected band migrating at 100 kDa, while it is observed for cells transfected with the PA plasmid.

PB2 (GTX125925, GeneTex, lot number 40926, 1:5000)
https://www.genetex.com/Product/Detail/Influenza-A-virus-PB2-protein-antibody/GTX125925
Validation data included in the manuscript: upon western blot analysis, lysates from control mock-transfected cells do not show the expected band migrating at 100 kDa, while it is observed for cells transfected with the PB2 plasmid.

ANP32A (AV40203, Sigma, lot number QC9984, 1:2500)
https://www.sigmaaldrich.com/FR/en/product/sigma/av40203
Validation data included in the manuscript: upon western blot analysis, lysates from ANP32AB-knock-out cells do not show the expected band migrating at 35 kDa, while it is observed in control cells.

ANP32B (EPR14588, AbCam, lot number GR3227402-4, 1:2500)
https://www.abcam.com/products/primary-antibodies/phapi2--april-antibody-epr14588-ab200836.html
Validation data included in the manuscript: upon western blot analysis, lysates from ANP32AB-knock-out cells do not show the expected band migrating at 35 kDa, while it is observed in control cells.

HRP-tagged secondary antibodies (Jackson Immunoresearch)

Validation

Validation of each primary antibody is described above

Eukaryotic cell lines

Policy information about cell lines and Sex and Gender in Research

Cell line source(s)

The 293T cells were purchased at ATCC (CRL-3216)
The 293T CTRL and ANP32AB KO cells were derived from 293T/17 cells purchased at ATCC (CRL-11268).
The MDCK cells were provided by the National Influenza Center, Paris, France.

Authentication

293T and 293T/17 cells were authentified by ATCC using STR profiling. Sex = female

Authentication	MDCK cells were not authentified
Mycoplasma contamination	Each cell line used has been tested on a regular basis for the absence of mycoplasma, using a specific PCR detection protocol. All cell lines tested negative for mycoplasma contamination
Commonly misidentified lines (See ICLAC register)	No commonly misidentified cell lines were used in the study

Plants

Seed stocks	N/A
Novel plant genotypes	N/A
Authentication	N/A
